# Supplementary material for: Adiabatic versus non-adiabatic electron transfer at 2D electrode materials
Source: Nat Commun. 2021 Dec 7;12:7110. doi: 10.1038/s41467-021-27339-9 (PMC8651748; doi:10.1038/s41467-021-27339-9)
Supplement: Supplementary file 1 — Supplementary Information [file 41467_2021_27339_MOESM1_ESM.pdf]

Supplementary Information

Adiabatic versus Non-Adiabatic Electron Transfer at 2D Electrode Materials

Dan-Qing Liu,[1,2] Minkyung Kang,[1,3] David Perry,[1] Chang-Hui Chen,[1] Geoff West,[4] Xue Xia,[5] Shayantan Chaudhuri,[1,6] Zachary P. L. Laker,[5] Neil R. Wilson,[5] Gabriel N. Meloni,[1] Marko M. Melander,*[,7] Reinhard J. Maurer,*[,1] and Patrick R. Unwin*[,1]

[1]*Department of Chemistry, University of Warwick, Coventry CV4 7AL, UK*

[2]*School of Materials Science and Engineering, Zhejiang University, Hangzhou, 310007, China*

[3]*Institute for Frontier Materials, Deakin University, Geelong, VIC 3217, Australia*

[4]*Warwick Manufacturing Group, University of Warwick, Coventry CV4 7AL, UK*

[5]*Department of Physics, University of Warwick, Coventry CV4 7AL, UK*

[6]*Centre for Doctoral Training in Diamond Science and Technology, University of Warwick, Coventry CV4 7AL, UK*

[7]*Department of Chemistry, Nanoscience Center, P.O. Box 35 (YN) FI-40014, University of Jyväskylä, Finland*

**Corresponding authors. Email address: marko.m.melander@jyu.fi,*

r.maurer@warwick.ac.uk, p.r.unwin@warwick.ac.uk

Contents

Supplementary Note 1: FE-SEM images of graphene on Cu foil 3

Supplementary Note 2: Raman spectra and mapping of graphene areas 1 and 2...... 4

Supplementary Note 3: Representative CVs extracted at pinholes in area 1...... 6

Supplementary Note 4: SECCM CVs on electropolished Cu foil 7

Supplementary Note 5: Supplementary kinetic analysis of areas 1 and 2...... 8

Supplementary Note 6: Extraction of kinetic parameters from SECCM images 9

Supplementary Note 7: Finite element method simulations...... 11

Supplementary Note 8: FE-SEM images of the end of pipet and corresponding scanned area 2...... 15

Supplementary Note 9: EBSD images of areas 1 and 2 16

Supplementary Note 10: Computational details of Density Functional Theory calculations 17

Supplementary Note 11: DOS and band structure of graphene-functionalized Cu(111) in vacuum...... 20

Supplementary Note 12: Dependency on unit cell 21

Supplementary Note 13: Newns-Anderson model of reduction reaction at electrochemical interface 24

13.1. Potential energy surface...... 24

13.2. Analyzing the rates using the electrochemical Newns-Anderson model...... 27

13.3. Conclusions from the Newns-Anderson model 33

Supplementary Note 14: KPFM characterization of characteristic graphene sample on Cu foils...... 34

Supplementary Note 15: DC ion conductance current histogram distributions of area 1 35

Supplementary References 36

Supplementary Note 1: FE-SEM images of graphene on Cu foil

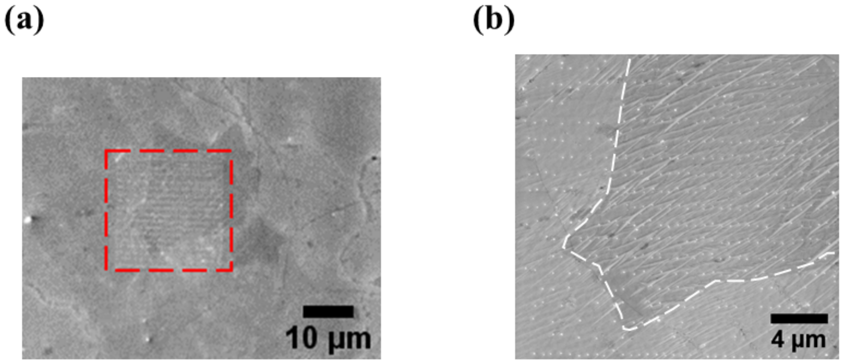

Supplementary Figure 1. (a) Wide area FE-SEM image of graphene on Cu foil, showing area 1 where SECCM measurements were performed, indicated by the red box. (b) FE-SEM image of the scanned area 1.

Supplementary Note 2: Raman spectra and mapping of graphene areas 1 and 2

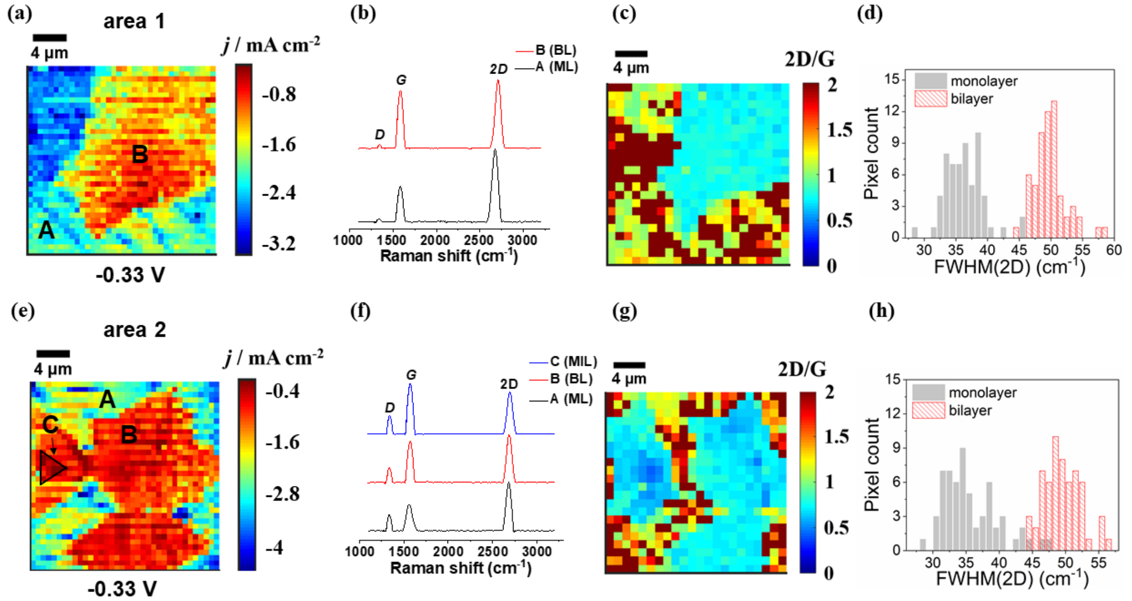

Supplementary Figure 2. (a) SECCM image of the reduction of $[Ru(NH_3)_6]^{3+}$ in area 1 at -0.33 V vs. Ag/AgCl. (b) Representative Raman spectra from regions A and B of area 1. (c) 2D/G ratio Raman map of area 1. (d) FWHM (2D) values from ML graphene (N = 60) and BL graphene (N = 60) of area 1. (e) SECCM image of the reduction of $[Ru(NH_3)_6]^{3+}$in area 2 at -0.33 V vs. Ag/AgCl. (f) Raman spectra from A, B and C of area 2. (g) 2D/G ratio Raman map of area 2. (h) FWHM (2D) values from ML graphene (N = 60) and BL graphene (N = 60) of area 2.

Raman mapping (spatial resolution of 1 µm) was executed on two different graphene regions, labeled A and B in area 1, categorized as zones with distinctly different current values in the corresponding SECCM images. Supplementary Figure 2b shows three characteristic graphene peaks for the D, G, and 2D bands.[1] The I_{2D}/I_G ratios of 2.1 (A) and 1.2 (B) indicate that the corresponding number of graphene layers are 1 and 2, respectively. The position of the 2D peak for A sits at 2672 cm^{-1}, while the B has an up-shifted 2D peak with respected to the A, which further indicates that A and B correspond to monolayer (ML) and bilayer (BL) graphene, respectively.[1,2] These data allowed for a map of the I_{2D}/I_G

ratio to be generated. There is a clear correlation between this Raman map (Supplementary Figure 2c) and the SECCM image in Figure 2b, indicating that the electrode current (activity) correlates with the number of graphene layers. The full width half-maximum (FWHM) values of ML and BL graphene in area 1 were analyzed by choosing 60 pixels randomly in each graphene region. The histogram in Supplementary Figure 2d indicates ML and BL graphene have average FWHM (2D) values of ~35 cm^{-1} and ~49 cm^{-1}, respectively, with the latter being a composite of 4 overlapping peaks.[1,3] The values are comparable with the Raman analysis reported for layer-by-layer stacked graphene[4] and graphene on Si/SiO_2.[3]

For area 2, a similar difference between the I_{2D}/I_G ratio is observed in regions A and B, corresponding to ML and BL graphene, respectively. However, the I_{2D}/I_G ratio for C is 0.83, suggesting that C represents multilayered (ML) graphene ($3 \leq$ layer number < 5 due to the self-limiting graphene growth on Cu).[5] Again, 2D/G ratio Raman mapping of area 2 correlated with the SECCM image in Figure 3a, as discussed in the main text. The FWHM (2D) values of area 2 are consistent with area 1.

Supplementary Note 3: Representative CVs extracted at pinholes in area 1

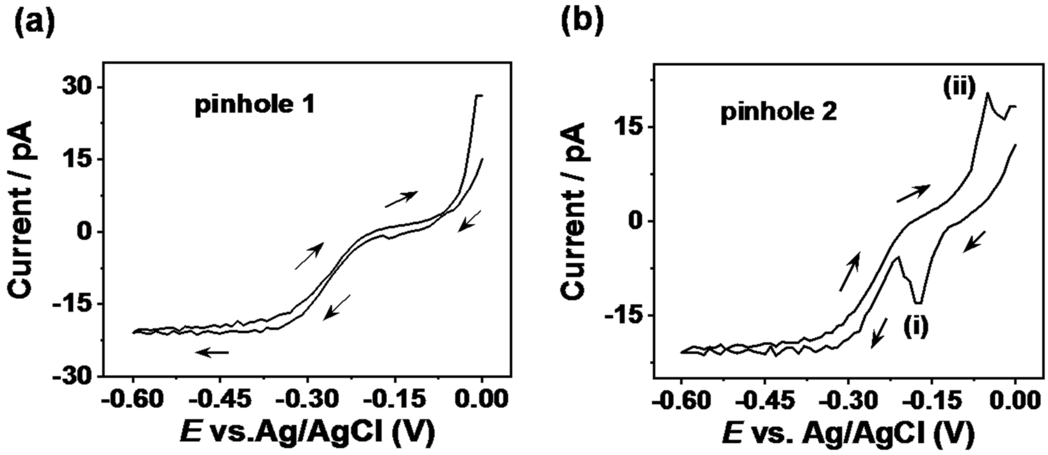

Supplementary Figure 3. (a) and (b) CVs extracted at pinhole 1 and pinhole 2 marked in Figure 2a. The aqueous solution in the SECCM tip contained 1 mM $[Ru(NH_3)_6]^{3+}$ in 50 mM KCl and the scan rate was 0.5 V/s.

The CV of pinhole 1 displays a positive current in the applied range 0 V to -0.05 V, attributed to the oxidation of Cu to Cu^+ ($CuCl_2^-$).[6,7] This is superimposed on a well-defined sigmoidal voltammogram for $[Ru(NH_3)_6]^{3+}$one-electron reduction to $[Ru(NH_3)_6]^{2+}$ at more cathodic potentials, as expected for SECCM.[3,5,8] The CV of pinhole 2 (see Supplementary Figure 3b) reveals a pair of more significant reduction and oxidation (i/ii) peaks superimposed on the $[Ru(NH_3)_6]^{3+}$ reduction wave.

Supplementary Note 4: SECCM CVs on electropolished Cu foil

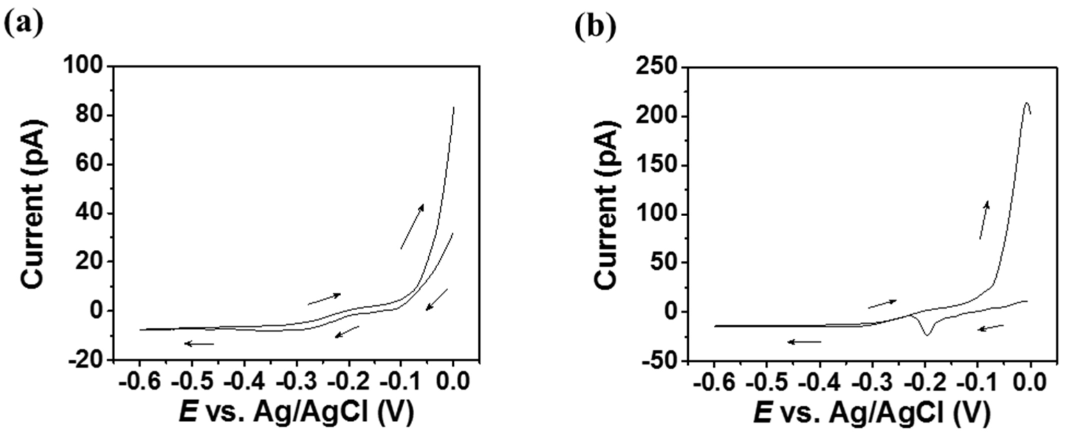

Supplementary Figure 4: Two SECCM spot CVs (different locations) for the reduction of 1 mM $[Ru(NH_3)_6]^{3+}$ in 50 mM KCl at 0.5 V/s run on electropolished Cu foil.

To further validate that the current signatures were from exposed Cu, SECCM CVs were run separately on a Cu foil after electropolishing (the same procedure as used to prepare the Cu foil for graphene growth), with the same SECCM parameters (Supplementary Note 3). The CVs exhibited similar characteristics to the pinholes in graphene. While SECCM may allow the size of pinholes in thin barriers on electrodes to be determined,[9] the purpose herein was to identify pinholes so that they could be ignored in any analysis, ensuring that only the electrochemistry of graphene was examined.

Supplementary Note 5: Supplementary kinetic analysis of areas 1 and 2

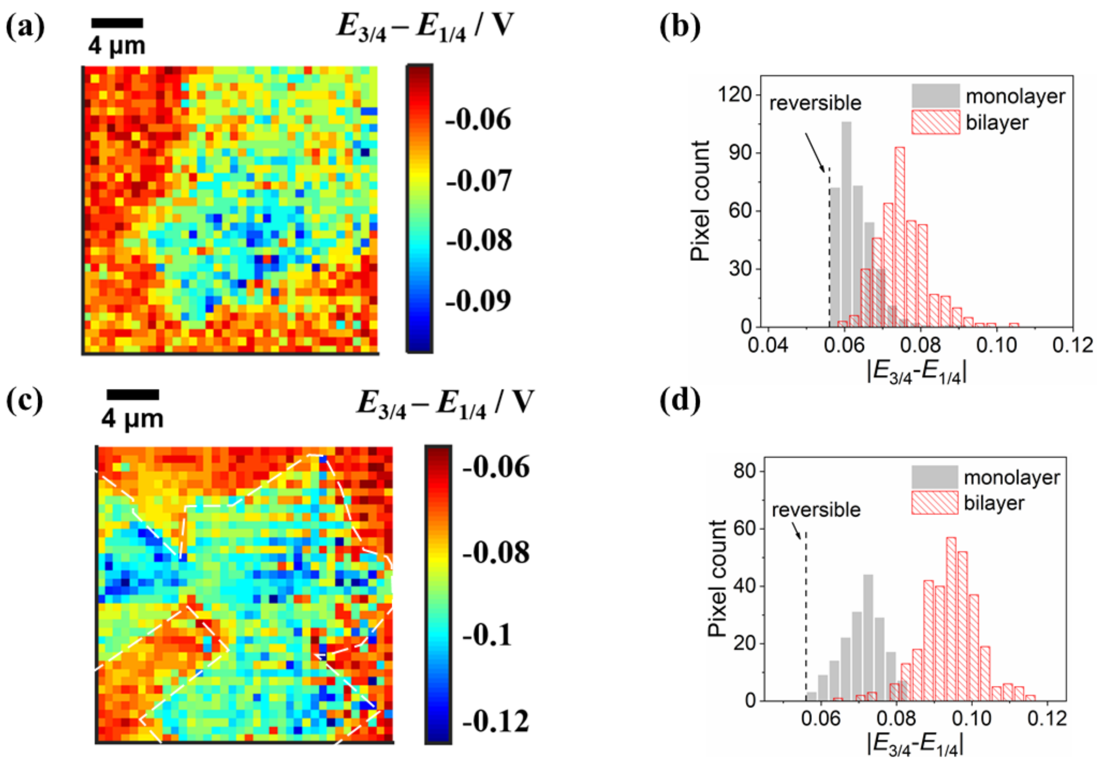

Supplementary Figure 5. (a) $|E_{3/4} – E_{1/4}|$ map of area 1, where the potential values are those at ¾ and ¼ of the maximum steady-state diffusion-limited current. (b) $|E_{3/4} – E_{1/4}|$ values on ML graphene (N = 355) and BL graphene (N = 404) of area 1. (c) $|E_{3/4} – E_{1/4}|$ map of area 2. (d) $|E_{3/4} – E_{1/4}|$ values on ML graphene (N = 176) and BL graphene (N = 308) of area 2.

Supplementary Note 6: Extraction of kinetic parameters from SECCM images

Here, we seek to understand the relative rate of electron transfer (ET) at ML vs. BL graphene at a particular potential, for comparison with rate theory. We have selected -0.33 V vs Ag/AgCl QRCE as the potential for the kinetic comparison, as this corresponds to a value either side of the half-wave potential for ML and BL graphene, from which the kinetic analysis can be made with good sensitivity and simply.

The formal potential $E^{0'}$ (-0.231 V vs. Ag/AgCl QRCE) was determined from SECCM measurements on a gold substrate using the same conditions as for the graphene on Cu foil (see Supplementary Figure 6). Since SECCM measurements are made under close to steady-state conditions, and the ML and BL graphene voltammograms are at an overpotential of ca. 100 mV with respect to the reversible case (see for example, Figure 2e and Figure 3e), it is reasonable to consider only the forward (reduction) process and neglect the back process (for the conditions outlined above). This simplifies the analysis and allows the direct calculation of the ratio of ET kinetics at ML and BL graphene, without any preconceptions as to the potential dependence of ET (*e.g.* no requirement to invoke Butler-Volmer kinetics or any assumptions about the value of the transfer coefficient). Under steady-state conditions, mass transport of the redox species (bulk concentration, c^*) to the electrode and the ET kinetics are in balance:

$$k_{\mathrm{T}}(c^* - c_{\mathrm{s}}) = k_{\mathrm{ET}} c_{\mathrm{s}} \qquad (1)$$

where k_T and k_{ET} are rate coefficients for mass transport and electron transfer, respectively, and c_s is the near-electrode surface concentration, related

to the steady-state current density by:

$$\frac{c_s}{c^*} = \frac{i_{\mathrm{lim}} - i}{i_{\mathrm{lim}}} \qquad (2)$$

where *i* is the current density and i_{lim} is the transport-limited current density. It follows that:

$$\frac{k_{\mathrm{T}}}{k_{\mathrm{ET}}+k_{\mathrm{T}}} = \frac{i_{\mathrm{lim}}-i}{i_{\mathrm{lim}}} \qquad (3)$$

Since:

$$k_{\mathrm{T}} = i_{\mathrm{lim}}/(nFc^*) \qquad (4)$$

where *n* is the number of electrons in the reaction and *F* is the Faraday constant, we obtain the following expression for k_{ET}:

$$k_{ET} = \frac{i}{nFc^*(1-i/i_{\mathrm{lim}})} \qquad (5)$$

and hence the ratio of ET kinetics at the ML (ML) to BL (BL) graphene at a particular potential is:

$$\frac{k_{\mathrm{ML}}}{k_{\mathrm{BL}}} = \frac{i_{\mathrm{ML}}(1 - i_{\mathrm{BL}}/i_{\mathrm{lim}})}{i_{\mathrm{BL}}(1 - i_{\mathrm{ML}}/i_{\mathrm{lim}})} \qquad (6)$$

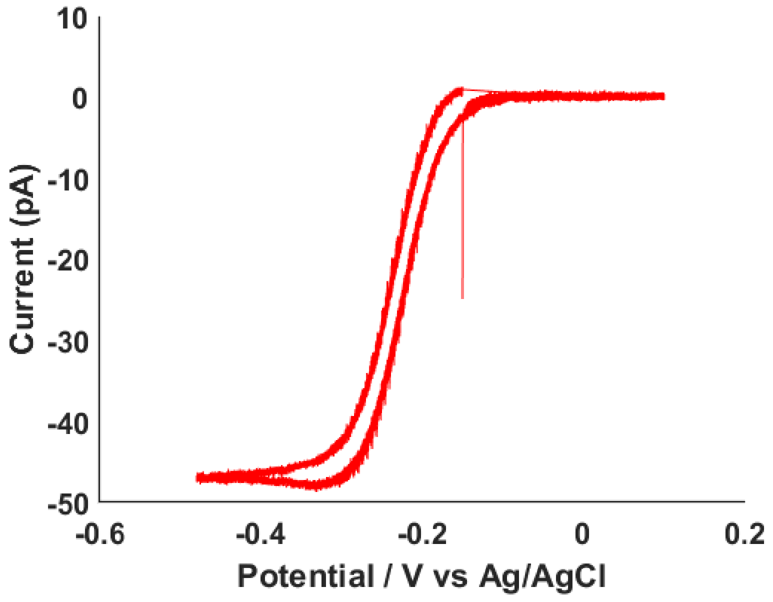

Supplementary Figure 6. CV of 1 mM $[Ru(NH_3)_6]^{3+}$ reduction on a gold substrate

in 50 mM KCl using the SECCM setup in Figure 1. The scan rate was 0.5 V/s.

Supplementary Note 7: Finite element method simulations

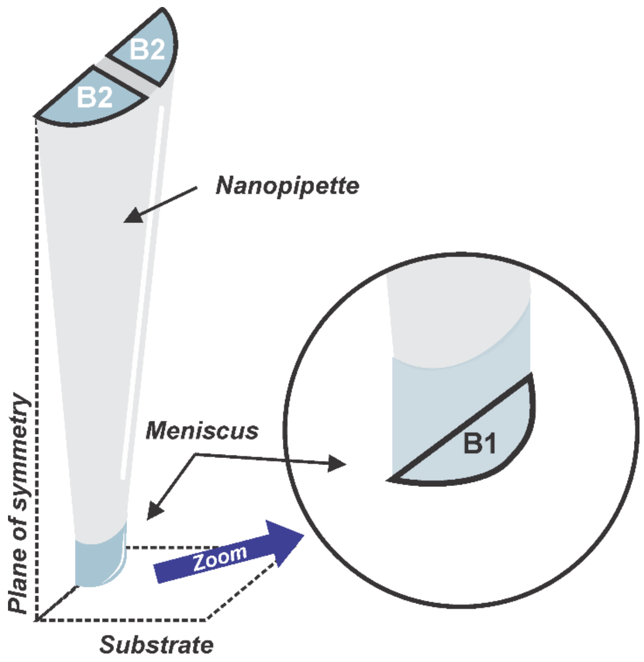

Supplementary Figure 7. Schematic representation of the 3D simulation domain used for the FEM model. Boundary conditions are specified in Supplementary Table 1. Drawing not to scale.

Supplementary Table 1. Summarized boundary conditions for the FEM model.

Boundary	Flux / Concentration condition
B1	$\boldsymbol{n} \cdot \boldsymbol{J}_{[\mathrm{Ru(NH3)_6}]^{3+}} = -(k_{Red}[\mathrm{Ru(NH_3)_6}]^{3+} - k_{Oxi}[\mathrm{Ru(NH_3)_6}]^{2+})]$
	$k_{Oxi} = k^0 \exp\left[(1-\alpha)\frac{F}{RT}\left(E_{app}(t) - E^{0'}\right)\right]$
	$k_{Red} = k^0 \exp\left[(-\alpha)\frac{F}{RT}\left(E_{app}(t) - E^{0'}\right)\right]$
B2	$[[\mathrm{Ru(NH_3)_6}]^{3+}] = 1\ \mathrm{mM}$

Supplementary Table 2. List of parameters used for FEM simulations

Symbol	Value	Description
$\boldsymbol{n}$	1	Number of electrons for $Ru(NH_3)_6^{3+}$ reduction
$\boldsymbol{\alpha}$	0.3 to 0.7	Transfer coefficient for the electrochemical process
$\boldsymbol{k^0}$	10^{-5} to 0.1 cm s^{-1}	Standard rate constant
$\boldsymbol{E^{0'}}$	0.231 V	Formal potential of the charge transfer reaction

E_{app}	0.2 to -0.5 V	Potential range applied to the working electrode
ν	0.5 V s^{-1}	Potential scan rate at the working electrode

To calculate values of k^0 and alpha (α) from the experimental data for the different graphene regions, linear sweep voltammograms (LSVs) were simulated for the SECCM configuration. All simulations were performed using COMSOL Multiphysics (v 5.6) and the transport of diluted species module. The simulation domain geometry, portrayed in Supplementary Figure 7 is representative of the nanopipette geometry (Supplementary Figure 10) and wetted area, which was considered to be the same size as the end of the tip internal dimensions for the reasons outlined in the main paper. A 3D model geometry was used to capture the theta pipette geometry (oval lumen and septum), with a symmetry plane running along the pipette length and perpendicular to the septum, to reduce computational cost. All boundaries within the simulation domain are described in Supplementary Figure 7 and Supplementary Table 1. The domain size (pipette height = 1.5 mm) and mesh density (> 350,000 elements) were sufficient to ensure accurate simulation results (LSVs). Mass transport was described by diffusion only to minimise the computational cost for the large number of simulations (*vide infra*). Although the bias between the two pipette barrels enhances the SECCM working electrode current through a migration component to the mass transport, for charged redox species like $[Ru(NH_3)_6]^{3+/2+}$, the effect is quite small at the small experimental bias used (200 mV).[8] To account for the enhanced mass transport, an effective diffusion coefficient for the ruthenium species was used.[3] A value of 8.8×10^{-6} cm^2 s^{-1} was found by matching simulated LSV limiting currents for a close to reversible process (k^0 = 1 cm s^{-1} and α = 0.5) to the experimental LSV at a gold substrate (Supplementary Figure 6). The potential-dependent flux for the 1-electron reduction of $[Ru(NH_3)_6]^{3+}$ at the substrate electrode (boundary B1 in Supplementary Table 1) was described by

Butler-Volmer kinetics with the potential-dependent oxidation and reduction rates described in Supplementary Table 1.

LSVs were simulated by sweeping the driving potential (E_{app}) linearly over time with the same voltammetric scan rate (0.5 V s^{-1}) as for the experimental studies. The formal potential ($E^{0'}$) was assumed to be the half-wave potential of $[Ru(NH_3)_6]^{3+}$ reduction at a gold electrode in the SECCM setup (Supplementary Figure 6). A series of 30 different LSVs (each containing 500 data points) was simulated for k^0 values ranging from 0.1 to 1 × 10 $^{-4}$ cm s^{-1}, and α, ranging from 0.3 to 0.7, with the tip geometry appropriate to the particular scan areas. Values of $E_{1/2}$ ($|E_{1/2}$ - E^{0}'|) and $E_{1/4}$ ($|E_{3/4}$ - $E_{1/4}|$) were calculated from the LSVs and used to construct a working surface (Supplementary Figure 8) correlating the kinetic parameters and the potential shifts.[10]

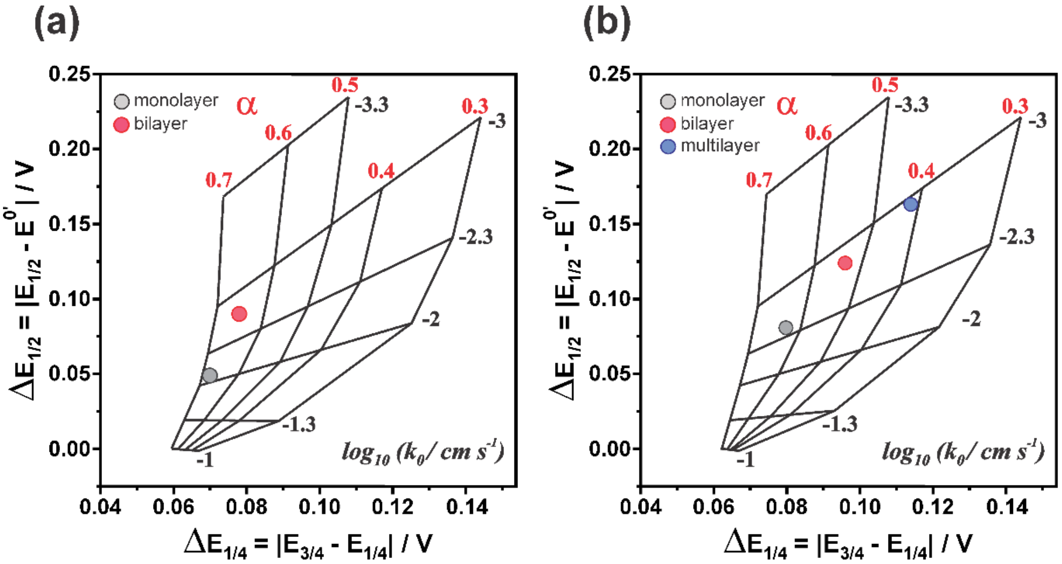

Supplementary Figure 8. Working surface constructed from $\Delta E_{1/2}$ ($|E_{1/2} - E^{0'}|$) and $\Delta E_{1/4}$ ($|E_{3/4} - E_{1/4}|$) values from simulated LSVs presented as values of $\log_{10}k^0$ and α for (a) area 1 and (b) area 2. Experimental potential shifts for ML (grey dot), BL (red dot) and multilayer (blue dot) graphene are overlaid on the working surface.

The experimental $E_{1/2}$ and $E_{1/4}$ for the average SECCM response at ML and BL graphene (area 1; main text Figure 2d) and at ML, BL and MlL graphene

(area 2; main text Figure 3c) were used to calculate k^0 and α values for these regions using the working surfaces in Supplementary Figure 8. The resulting values are summarized in Supplementary Table 3.

Supplementary Table 3. Kinetic parameters calculated by FEM for the two distinct graphene regions.

**Area 1**	**k^0 / cm s^{-1}**	**α**
ML	7.6×10^{-3}	0.66
BL	3.1×10^{-3}	0.63
**Area 2**		
ML	3.7×10^{-3}	0.61
BL	2.1×10^{-3}	0.52
MIL	1.4×10^{-3}	0.42

With the values in Supplementary Table 3, LSVs for each case were simulated for both areas (Supplementary Figure 9) and used to calculate the ratio of ET kinetics at the ML to BL graphene at a potential of -0.33 V, using equation 6. We calculate k_{ML}/k_{BL} = 3.3 (area 1) and 2.8 (area 2), commensurate to the calculated values in the main manuscript.

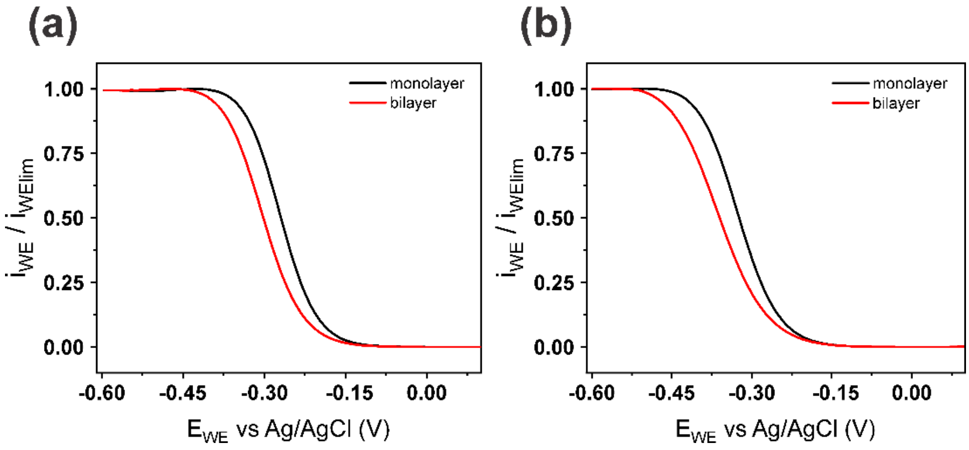

Supplementary Figure 9. Simulated LSVs for ML and BL graphene using the kinetic parameters from Supplementary Table 3 for (a) area 1 and (b) area 2.

Supplementary Note 8: FE-SEM images of the end of pipet and corresponding scanned area 2

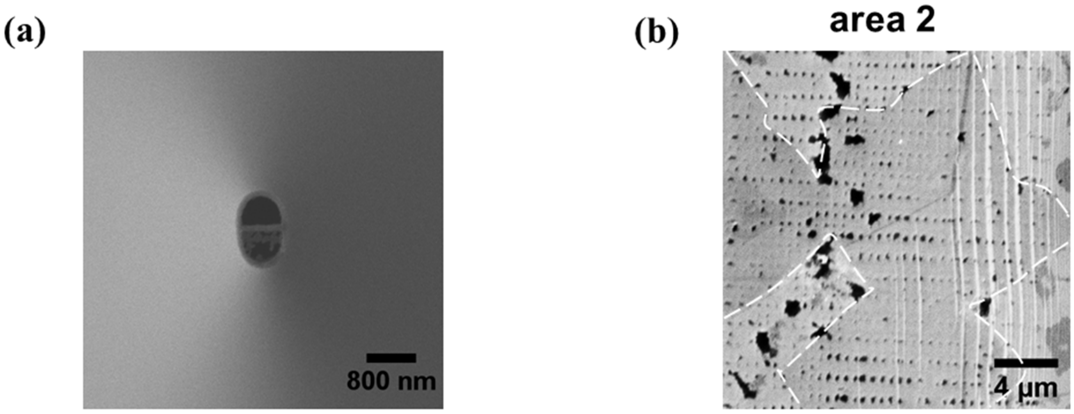

Supplementary Figure 10. (a) Typical FE-SEM image of the end of pipet used for scanning area 2. (b) Corresponding FE-SEM image of the scanned area 2.

Supplementary Note 9: EBSD images of areas 1 and 2

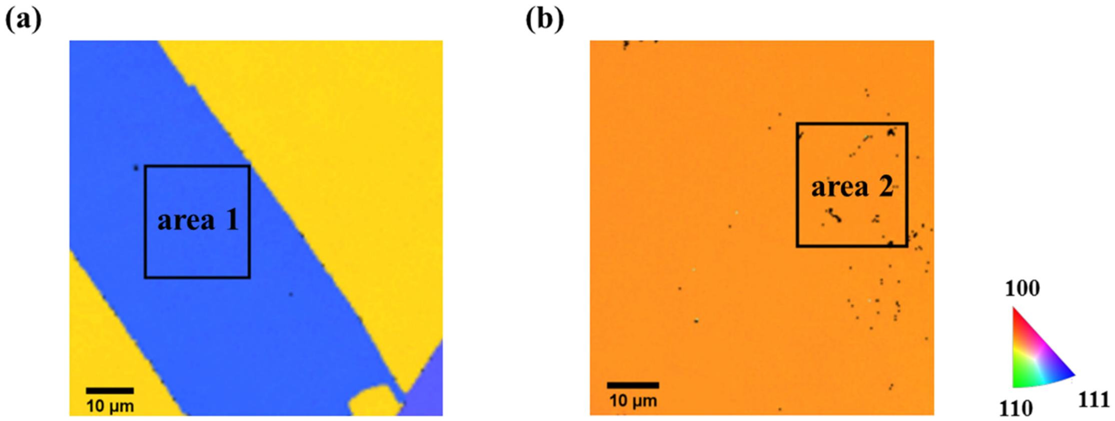

Supplementary Figure 11. EBSD images of (a) area 1 and (b) area 2. The orientation maps are color coded according to the growth direction.

Supplementary Note 10: Computational details of Density Functional Theory calculations

FHI-aims. Density functional theory (DFT) calculations were performed with the all-electron atomic-orbital code FHI-aims using the default *tight* basis set definition[11] and the GPAW code using the projector-augmented wave method.[12-15] We modeled the graphene adsorption on a Cu(111) surface using metal-screened dispersion-inclusive PBE functional (PBE+vdW^{surf}).[16] A primitive surface unit cell (lattice constant) slab with 30 Å of vacuum perpendicular to the surface. Geometries were optimized for the clean Cu(111) surface, and for one, two, three, and four stacked layers of graphene on Cu(111) up to a maximum force of 0.01 eV/Å with a Monkhorst-Pack k-grid sampling of 12×12×1.

GPAW. The DFT calculations were performed within the projector-augmented wave method[12] as implemented in the GPAW code.[13-15] The local double-zeta polarized basis[17] and a grid spacing of 0.2 Å were used. The systems were modelled as 2D slabs periodic in xy-directions and k-points were sampled using a 4×4×1 grid. 15 Å of vacuum was inserted on both sides of the slabs. Note that the real-space GPAW code enables truly 2D calculations without any periodicity of the z-direction (in the [111] direction). The exchange-correlation effects were accounted for using the PBE functional[18] with the vdW^{surf} approach[16] to account for van der Waals interactions. The original molecular Tkatchenko-Scheffler vdW parameters[16] were used for H, N, C, and Ru while the revised vdW^{surf} parameters for metals[16] were used for Cu. All calculations were fully relaxed until the residual force was below 0.05 eV/Å.

The model systems representing experiments were based on 4×4×5

Cu(111) slabs. The ML and BL graphene-Cu were obtained by depositing one, two, or three layers of graphene on Cu, as shown in Figure 4 of the main article. A single graphene layer consists of 32 carbon atoms. To model the Cu-graphene systems under electrochemical conditions, calculations were performed within the constant potential, grand-canonical DFT (GC-DFT) formalism.[19] GC-DFT calculations were performed using the solvent jellium method (SJM)[20] by modifying the number of electrons until the Fermi level matches the specified value*.* Dirichlet zero boundary conditions detailed in Supplementary Ref. [19] were implemented and used for solving the electrostatic potential to obtain a well-defined absolute reference potential within the SJM (see below). The liquid environment was taken into account by using a continuum solvent model[21] for water.

Using the GC-DFT/SJM approach, the Cu-graphene were simulated at two potentials: the potential of zero charge (PZC) and -330 mV vs. Ag/AgCl QRCE (for the reasons outlined above). The PZC calculations were realized using a net zero charge for the calculations. The computational reference potential was determined by computing the zero charge Fermi-level for the pure copper slab which was referenced against the experimental PZC of Cu(111)[22] to provide a connection between the computational and experimental PZC. This yields an absolute, single electrode potential[23] following the procedure detailed in equations 28-32 of Supplementary Ref. [19]. Supplementary Table 4 shows the PZC and the surface potential ${}_{\mathrm{S}}\Delta_{\mathrm{M}}\psi$ (defined as the difference between absolute electrode potential [4th column in Supplementary Table 4] in contact with solvent and metal work function [3rd column in Supplementary Table 4[24]]). As can be

seen in the last column, our calculations predict that the surface potential of BL graphene on copper is 210 meV higher than that of ML graphene on copper. This is in qualitative agreement with experimental Kelvin Probe Microscopy Measurements (KPFM) conducted in this study. (See Supplementary Note 14).

Supplementary Table 4. Potential of Zero Charge (PZC), dipole-corrected gas-phase work function, dipole-corrected solvent-exposed work function, and surface potential $s\Delta_M\psi$ for a clean Cu(111) surface and ML, BL, and trilayer graphene on Cu(111).

System	PZC in eV	Dipole-layer corr. Workfunction gas in eV	Dipole-layercorr. Workfunction solvent in eV	$s\Delta_M\psi$ in eV
Cu(111)	4.17	4.18	4.17	-0.01
ML graphene	3.60	3.88	3.31	-0.28
BL graphene	4.02	4.09	3.95	-0.07
Trilayer graphene	4.24	4.20	4.28	0.04

Supplementary Note 11: DOS and band structure of graphene-functionalized Cu(111) in vacuum

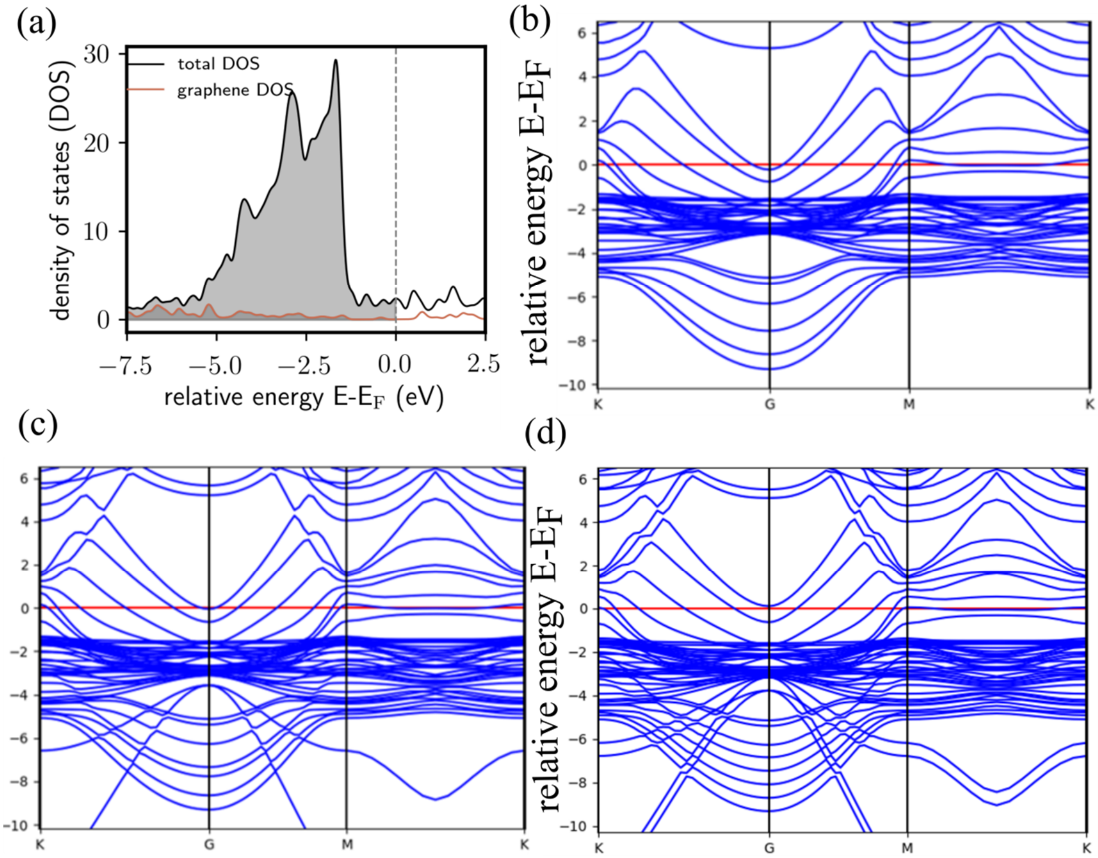

Supplementary Figure 12. (a) Density-of-States (DOS) of ML graphene on Cu(111) in vacuum. The dashed vertical line depicts the Fermi level. The shaded area depicts occupied states. (b) Band structure of clean Cu(111) surface. Red horizontal line indicates the Fermi level. (c) Band structure of ML graphene on Cu(111). (d) Band structure of BL graphene on Cu(111).

Supplementary Note 12: Dependency on unit cell

To ensure the unit cell was not a limiting factor in the results and conclusions reached, calculations were repeated using a smaller, rectangular unit cell; Supplementary Fig. 13 depicts both unit cells that were used.

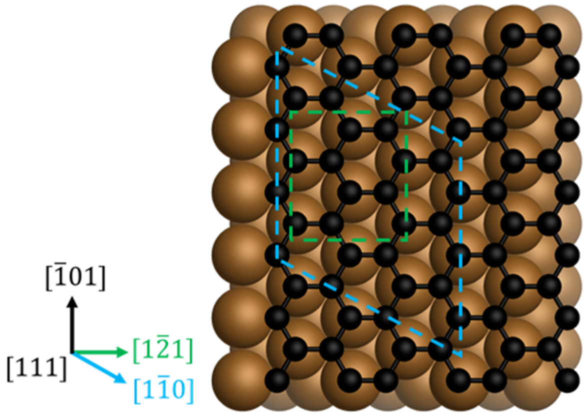

Supplementary Figure 13. Top view of graphene adsorbed on Cu(111), rendered using PyMOL. Carbon and copper atoms are shown in black and brown respectively, with the small and large unit cells shown in green and blue respectively.

We find that our results only weakly depend on the unit cell choice. As can be seen in both Fig. 4(c) and (d), and Supplementary Figure 14, the graphene states do not significantly contribute to the DOS around the Fermi level for either unit cell, and there is no discernible difference between the DOS plots. The Volta potential was found to be 0.14 V with the small unit cell as opposed to 0.21 V with the larger one (Supplementary Figure 15), and the electrostatic potential decay difference between mono- and BL graphene was found to be 0.05 eV and 0.06 eV at the height of the adsorbate for the small and large unit cells respectively (Supplementary Figure 16).

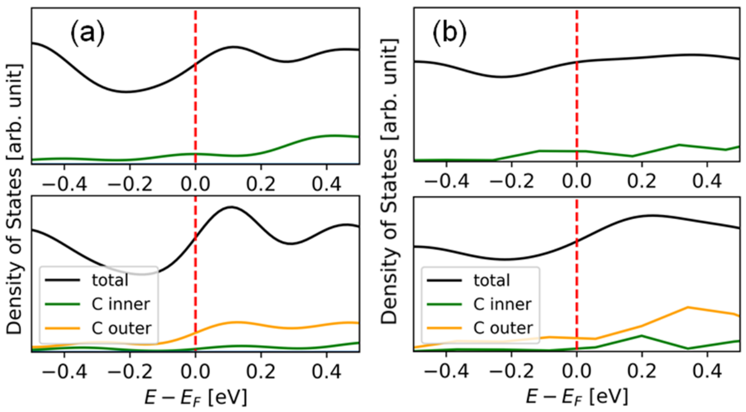

Supplementary Figure 14. Density of states centered around the Fermi level (E_F) for ML (top) and BL (bottom) graphene on Cu(111) for the (a) large and (b) small unit cells, calculated at a fixed external potential of –0.33 V vs. Ag/AgCl QRCE. Shown are the total DOS and the projected DOS for graphene. The red dashed line corresponds to the Fermi level.

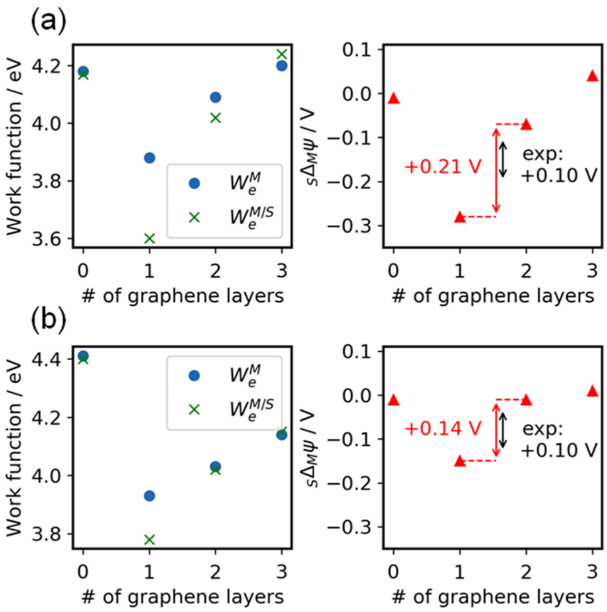

Supplementary Figure 15. (Left) W_e^M (blue circles) and $W_e^{M/S}$ (green crosses) as a function of number of graphene layers, and (Right) Contact potential as a function of graphene layers compared against the ML/BL contact potential difference measured by KPFM (red: calculation, black: KPFM measurement) for (a) the large unit cell and (b) the small unit cell.

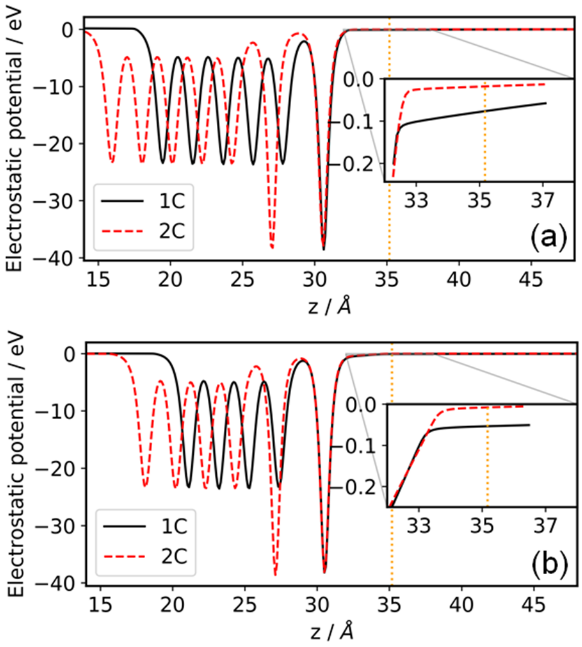

Supplementary Figure 16. Electrostatic potential of ML (1C) and BL (2C) graphene on Cu(111) in the direction perpendicular to the electrode, calculated at a fixed external potential of –0.33 V vs. Ag/AgCl QRCE for the (a) large and (b) small unit cells. The dashed orange line denotes the position of the Ru atom in $[Ru(NH_3)_6]^{3+}$ and corresponds to the height *d* that enters the model Hamiltonian.

Supplementary Note 13: Newns-Anderson model of reduction reaction at electrochemical interface

The electron transfer rate is given as

$$k_{red} = \kappa \times e^{-\frac{\Delta G^{\ddagger}}{k_B T}} \tag{7}$$

For adiabatic reactions $\kappa \approx \frac{k_B T}{h}$ and for non-adiabatic reactions, $\kappa \approx \frac{2\pi}{h}\frac{V^2}{\sqrt{4k_B T\lambda}}$, where λ is the reorganization energy and V is the coupling constant. To bridge between these two limits, the Landau-Zener formula[25] can be utilized. The activation energy is obtained using the Schmickler-Newns-Anderson (SNA) model described below.

13.1. Potential energy surface

The activation energy can be approximated using the Newns-Anderson Hamiltonian[26,27] adapted to the electrochemical setting by Schmickler.[28,29] This Hamiltonian can capture both the adiabatic and non-adiabatic limits depending on the coupling constant, V, and whether perturbation theory or Dyson series approach is used for the solution.[29] We have modified the original SNA model to account for the electrostatic interaction between the electrode and the redox coupled as done in Refs[30,31] for proton-coupled electron transfer reactions. Then, the Hamiltonian is

$$H = H_{el} + H_{sol} + H_{int} + H_{\phi} \tag{8}$$

The Hamiltonian depends on the following variables: z is the charge state of the oxidized redox couple ($z = 3$ for Ru(III) in $[Ru(NH_3)_6]^{3+}$), n is the occupation number of the redox orbital (0 for initial state, 1 for final state), q is the (solvent) reorganization coordinate, ε is the energy of the redox level, V_k is the interaction

parameter, p is solvent momentum, and ω is the solvent frequency. The electronic part is given as

$$H_{\text{el}} = \varepsilon(d)n + \sum_k \varepsilon_k n_k + \sum_k [V(d)_k c_k^+ c_a + V(d)_k^* c_a^+ c_k] \tag{9}$$

where d is the distance between the redox couple and the electrode. The solvent contribution for classical nuclei is

$$H_{\text{sol}} = \frac{1}{2}\hbar\omega(p^2 + q^2) \tag{10}$$

The interaction energy between the solvent and the reactant depends linearly on solvent coordinate and the coupling strength g

$$H_{\text{int}} = (z - n)\hbar\omega g q \tag{11}$$

As it was found that electrostatic potential ϕ of the ML system is much higher than that of the BL, we account for this via the electrostatic interaction between the redox molecule and the electrode as[30,31]

$$H_{\phi} = (z - n)\phi(d) \tag{12}$$

It is to be noted that $z\phi(d)$ presents a repulsive Coulombic interaction between the $[Ru(NH_3)_6]^{3+}$ redox center and the electrode. As such, $\phi(d)$ has the opposite sign than the electrostatic potential commonly obtained from electronic structure theory calculations where the electrostatic potential is seen from the electrons' perspective. The $(z - n)\phi(d)$ term denotes the change in the Coulombic interaction due to the reduction and the higher the $\phi(d)$, the larger the stabilization due to the reduction of Ru(III) to Ru(II).

Next the total Hamiltonian is rearranged by collecting all n dependent terms to give

$$H = \varepsilon'(d)n + \sum_k \varepsilon_k n_k + \sum_k [V(d)_k c_k^+ c_a + V(d)_k^* c_a^+ c_k] + H_{\text{sol}} + z\hbar\omega g q + z\phi(d)$$

(13)

where the effective redox state energy is

$$\varepsilon'(d) = \varepsilon - \hbar\omega g q - \phi(d) \quad (14)$$

By making use of the Hellman-Feynman theorem one obtains

$$\left\langle\frac{\partial H}{\partial q}\right\rangle = 0 \rightarrow (n - z)g = 0 \quad (15)$$

This can be utilized to write the reorganization energy as

$$\lambda = \frac{1}{2}\hbar\omega g^2 \quad (16)$$

With these definitions the Hamiltonian becomes

$$H = \varepsilon'(q,d)n + \lambda q^2 + 2\lambda q + \sum_k \varepsilon_k n_k + \sum_k [V(d)_k c_k^+ c_a + V(d)_k^* c_a^+ c_k] + z\phi(d) \quad (17)$$

where the effective redox state is given as

$$\varepsilon_a'(q,d) = \varepsilon_a(d) - 2\lambda(z - n) - \phi(d) \quad (18)$$

Additionally, as done in Ref [30], the electrode potential is accounted by shifting the energy of the electron at the Fermi-level yielding

$$H(q,d,E) = \varepsilon'(q,d,E)n + \lambda q^2 + 2\lambda q + \sum_k \varepsilon_k n_k + \sum_k [V(d)_k c_k^+ c_a + V(d)_k^* c_a^+ c_k] + $$
$$+ z\phi(d) - eE \quad (19)$$

and

$$\varepsilon'(q,d,E) = \varepsilon(d) - 2\lambda(z - n) - \phi(d) + eE \quad (20)$$

This is the final form of the Hamiltonian. Comparing these final expressions to the ones obtained in Supplementary Ref. [30] it is noticed that our final expression is equivalent, apart from the terms including z which are due to the higher oxidation state of Ru(III) than the $[H_3O]^+$studied in Supplementary Ref. [30].

In the general case, the electronic part can be solved using Green function techniques to yield the DOS of the redox center. This gives the DOS as in Supplementary Ref. [28-30]

$$\rho_{\mathrm{a}}(q,d,\varepsilon) = \frac{1}{\pi}\frac{\Delta(d,\varepsilon)}{\left(\varepsilon-\left(\varepsilon_{\mathrm{a}}'(q,d)+\Lambda(d,\varepsilon)\right)\right)^2+\Delta(d,\varepsilon)^2} \quad (21)$$

using the chemisorption functions

$$\Delta(d,\varepsilon) = \sum_k |V_k(d,\epsilon)|^2 \pi\delta(\varepsilon-\varepsilon_k) \approx \pi|V(d)|^2\rho(\epsilon) \quad (22)$$

$$\Lambda(d,\varepsilon) = \frac{1}{\pi}\mathcal{P}\int \frac{\Delta(d,\varepsilon')}{\varepsilon-\varepsilon'}\mathrm{d}\varepsilon' \quad (23)$$

where $\mathcal{P}$ denotes the principal value and $\rho(\epsilon)$ denotes the electrode's DOS. The k-independent, effective coupling constant is generally a good approximation, and results from the wide band approximation: $\Delta(d,\varepsilon) \approx \pi\Delta\rho(\varepsilon,d)$ and $\Lambda = 0$.[28-29] This approximation is justified given the structureless and constant nature of the density of states for both the ML and BL electrodes around the Fermi level. Following the treatment in Supplementary Refs. [28-30] for an analogous situation finally leads to the energy of the system

$$H(d,q,E) = \lambda q^2 + 2\lambda q + \varepsilon'(q,d,E)\bar{n}(q,d) + \frac{\Delta(d)}{2\pi}\ln\frac{\varepsilon'(q,d,E)^2+\Delta(d)^2}{\varepsilon_a^2+\Delta(d)^2} +$$

$$+z\phi(d) - eE \quad (24)$$

where the occupation number for the redox state is computed using

$$\bar{n}(q,d) = \int_{-\infty}^{\varepsilon_{\mathrm{f}}} \rho_{\mathrm{a}}(q,d,\varepsilon)\mathrm{d}\varepsilon \quad (25)$$

13.2. Analyzing the rates using the electrochemical Newns-Anderson model

As shown in Supplementary Figure 17, the total DOS (ρ) for both ML and BL graphene are almost identical. Also, the charge z, the distance d, λ, and q at initial, transition, and final states are the same for both systems. The redox orbital occupation $\bar{n}(q,d)$ at the initial and final states are equal for the studied systems. Below the adiabatic and non-adiabatic barriers are analyzed.

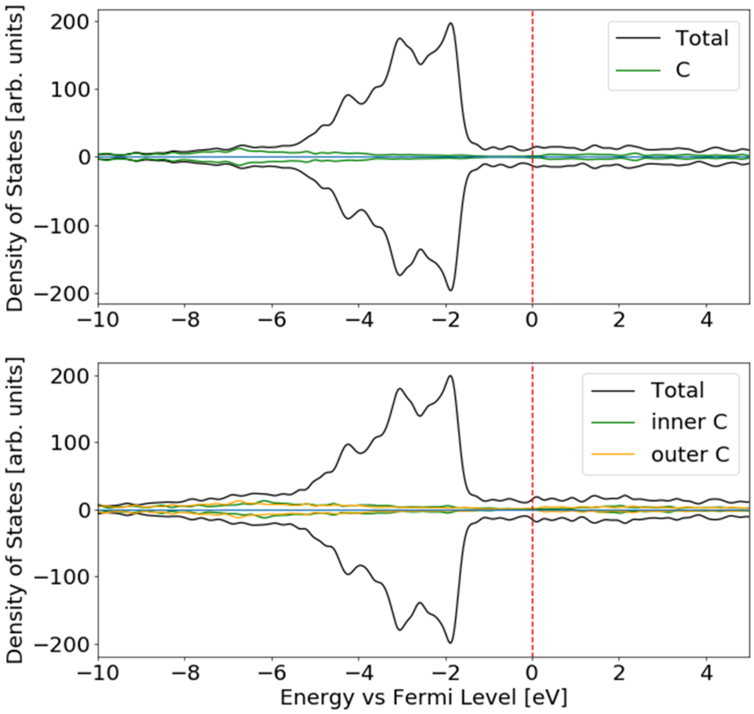

Supplementary Figure 17. Density of states (DOS) for ML (top) and BL (bottom) graphene on Cu(111) calculated at fixed external potential of -0.33 V vs. Ag/AgCl. Shown are the total DOS and the projected DOS for graphene. Red dashed line corresponds to Fermi level.

13.2.1 Adiabatic reactions

For adiabatic reactions the wide band coupling Δ is assumed to be equal for both systems and the DOSs of both systems are equal. As a result, the projected DOS (ρ_a) and hence the occupation number $\bar{n}$ are equal for both systems. The reaction barrier is given as

$$\Delta G^{\ddagger}_{adiabatic} = H(d, q^{\ddagger}, E) - H(d, q^{0}, E) \tag{26}$$

Since we are interested in the difference on the charge transfer kinetics of the ML and BL systems it is reasonable to focus on the difference in activation energy as for the adiabatic reaction:

$$r_{adiabatic} = \frac{k_{\mathrm{ML}}}{k_{\mathrm{BL}}} = \exp\left[-\frac{\Delta\Delta G^{\ddagger}_{adiabatic}}{k_B T}\right]. \tag{27}$$

The difference in activation energies is

$$\Delta\Delta G^{\ddagger}_{adiabatic} = \left(\varepsilon'^{,\ddagger}_{\mathrm{ML}}(q,d,E) - \varepsilon'^{,\ddagger}_{\mathrm{BL}}(q,d,E)\right)\bar{n}(q,d)$$

$$+\frac{\Delta(d)}{2\pi}\ln\frac{\left(\varepsilon'^{,\ddagger}_{\mathrm{ML}}\right)^2 + \Delta(d)^2}{\left(\varepsilon'^{,\ddagger}_{\mathrm{BL}}\right)^2 + \Delta(d)^2}$$

$$-\frac{\Delta(d)}{2\pi}\ln\frac{\left(\varepsilon'^{,0}_{\mathrm{ML}}\right)^2+\Delta(d)^2}{\left(\varepsilon'^{,0}_{\mathrm{BL}}\right)^2+\Delta(d)^2} \tag{28}$$

For large Δ, *i.e.* a strongly adiabatic reaction, the biggest contribution comes from the first term as the other two cancel. At the transition state $\bar{n} \approx 0.5$ is expected. Using these approximations for the equilibrium distance between the redox center and the electrode shown in Figure 5d, we obtain

$$\Delta\Delta G_{adiabatic}(d_{eq}) \approx 0.5 \times \left[-\phi_{\mathrm{ML}}(d_{eq}) - \left(-\phi_{\mathrm{BL}}(d_{eq})\right)\right]$$

$$= 0.5\left[\phi_{\mathrm{BL}}(d_{eq}) - \phi_{\mathrm{ML}}(d_{eq})\right]$$

$$\approx 0.5 \times (0.03. -0.1) = -0.035eV \tag{29}$$

This leads to

$$r_{adiabatic}(d_{eq}) \approx \exp\left[-\frac{-0.035eV}{k_B T}\right] \approx 4 \tag{30}$$

Displacing the Ru-center from the equilibrium position by 1Å closer to (further from) the electrode results in the rate ratio of 7.5 (2.7). Therefore, the predicted adiabatic rate ratio is robust against even rather large variations in the position of the redox couple.

13.2.2 Non-adiabatic reactions

The barrier for a non-adiabatic reaction is the same as for the adiabatic reaction as $\Delta \to 0$. The real difference arises for the prefactor in the rate expression. The

coupling constant V measures tunneling probability and exhibits an exponential dependence on the distance between the electron donor and acceptor. Then, the effective coupling term in Eq. 23 can be written as

$$V(d) \approx V(d_0)\exp[-\beta(d-d_0)] \quad (31)$$

where d_0 is a reference distance, and typically $\beta \in (0.1,3)$ Å^{-1} and values below 0.1 are considered exceedingly low.[25,32] The coupling constant for an outer-sphere couple can be estimated[33] as $V(d_0) \approx \langle \psi_e(d_0) | \hat{V}(d_0) | \psi_m(d_0) \rangle \approx \phi(d_0)\langle \psi_e(d_0) | \psi_M(d_0) \rangle$ in terms of an effective electrode orbital (ψ_e), the LUMO of the redox couple (ψ_m), the perturbation $\hat{V}$, and the coupling is directly proportional to the orbital overlap. The effective electrode orbital is obtained from the electron density decay $\rho(d) = |\psi_e(d)|^2 = \rho(d_0)\exp[-\beta(d-d_0)]$. Naturally, the LUMO for both ML and BL systems is equal. $\phi(d)$ is the position-dependent electrostatic potential which we obtain from constant potential DFT.

Next, we analyze the non-adiabaticity of $[Ru(NH_3)_6]^{3+}$ reduction on the ML and BL electrodes using the above model for the coupling constant. As seen from the total DOSs in Supplementary Figure 17, the highest energy orbitals reside mostly on the Cu atoms. This means that the term $\rho(\epsilon)$ in Eq. 22 is equal for both systems. Also, as shown in Supplementary Figure 18, the electron density near the redox center is slightly larger for the ML system but very similar for both systems. This strongly indicates that the effective electrode orbital ψ_e and as a consequence the overlap and coupling between electrode and $[Ru(NH_3)_6]^{3+}$ are the same for both systems. As a result, the possible differences in the ML/BL electrodes only arise from their local electrostatic potential differences.[34]

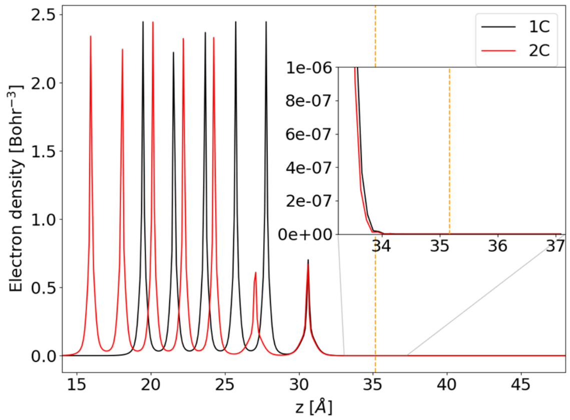

Supplementary Figure 18. Electron density for the ML (1C) and BL (2C) graphene electrodes as a function of the distance between the electrode surface calculated at a fixed electrochemical potential corresponding to -0.330 V vs. Ag/AgCl. The orange dotted line indicates the position of the redox couple.

Besides the coupling prefactor, the coupling constant also affects the barrier. For small V the coupling constant $\Delta \rightarrow 0$ and the diabatic $\Delta\Delta G^{\ddagger}_{diabatic}$ is

$$\Delta\Delta G^{\ddagger}_{diabatic} = \left(\varepsilon'^{,\ddagger}_{\mathrm{ML}}(q,d,E) - \varepsilon'^{,\ddagger}_{\mathrm{BL}}(q,d,E)\right)\bar{n}(q,d) \tag{32}$$

Pulling all threads together we can now evaluate the ratio between the non-adiabatic rates as

$$r_{diabatic}(d) = \frac{V^2_{\mathrm{ML}}(d_{eq})}{V^2_{\mathrm{BL}}(d_{eq})}\exp\left[-\frac{\Delta\Delta G(d)^{\ddagger}_{diabatic}}{k_B T}\right] = \frac{\phi^2_{\mathrm{ML}}(d)}{\phi^2_{\mathrm{BL}}(d)}\frac{\exp[-2\beta(d_{\mathrm{ML}}-d)]}{\exp[-2\beta(d_{\mathrm{BL}}-d)]}r_{adiabatic}(d)$$

$$= r_{adiabatic}(d)\frac{\phi^2_{\mathrm{ML}}(d)}{\phi^2_{\mathrm{BL}}(d)}\exp[2\beta(d_{\mathrm{BL}} - d_{\mathrm{ML}})] \tag{33}$$

where the coupling constant can be estimated using the local electrostatic potentials as the perturbation $V(d) \approx \phi(d)\langle\psi_e(d)|\psi_M(d)\rangle$ and using that $\langle\psi_e(d)|\psi_M(d)\rangle$ is equal for both ML and BL systems, as discussed above. The distances are taken as the separation between the copper and the redox centers

inferred from Supplementary Figure 18: $d_{\mathrm{BL}} \approx 10$ Å and $d_{\mathrm{ML}} \approx 7$ Å.

$$r_{diabatic}(d) = r_{adiabatic}(d)\frac{\phi_{\mathrm{ML}}^2(d)}{\phi_{\mathrm{BL}}^2(d)}\exp[6\beta] \tag{34}$$

Evaluating this expression at the equilibrium distance using the values from electrostatic potentials obtained from constant potential DFT gives

$$r_{diabatic}(d_{eq}) \approx 4\exp[6\beta]\frac{\phi_{\mathrm{ML}}^2(d_{eq})}{\phi_{\mathrm{BL}}^2(d_{eq})} = 4\exp[6\beta]\frac{0.03^2}{0.1^2} \approx 0.36\exp[6\beta]$$

$$\approx \begin{cases} 2.5 \text{ for } \beta = 0.1\text{Å}^{-1} \\ 29 \text{ for } \beta = 0.5\text{Å}^{-1} \\ 576 \text{ for } \beta = 1.0\text{Å}^{-1} \end{cases} \tag{35}$$

We can account for the variations in the redox couple's position by displacing the Ru-center from the equilibrium position by 1Å closer to (further from) the electrode. This results in the rate ratios

$$r_{diabatic}(d_{eq} - 1) \approx \begin{cases} 1 \text{ for } \beta = 0.1\text{Å}^{-1} \\ 11 \text{ for } \beta = 0.5\text{Å}^{-1} \\ 225 \text{ for } \beta = 1.0\text{Å}^{-1} \end{cases}$$

$$r_{diabatic}(d_{eq} + 1) \approx \begin{cases} 0.4 \text{ for } \beta = 0.1\text{Å}^{-1} \\ 5 \text{ for } \beta = 0.5\text{Å}^{-1} \\ 98 \text{ for } \beta = 1.0\text{Å}^{-1} \end{cases} \tag{36}$$

These results show that the non-adiabatic rates are very sensitive to both the decay constant β and the distance between the surface and the redox couple. Note that the absolute rates are dominated by the smaller distances and hence tunneling probability decreases quickly as the distance increases making the equilibrium and closer-to-the-surface cases more relevant for the overall rate.

13.3. Conclusions from the Newns-Anderson model

The ratio of the experimentally measured rates - main text: k_{ML}/k_{BL} = 4.37 (area 1) and 4.22 (area 2) is very similar to the theoretically approximated value for adiabatic reactions at the equilibrium distance ($r_{adiabatic}(d_{eq}) \approx 4$) which results from the difference in the electrostatic potentials of the ML and BL systems. We also showed that the adiabatic rate constant ratio is robust against variations in the redox couple's position. By including the non-adiabaticity and accounting for the electrostatics in the reaction barrier as well as the perturbation term in the coupling matrix element, a good match with experiments is obtained with exceedingly small values of $\beta \approx 0.1$ Å^{-1} for the equilibrium redox couple position.

Based on the above considerations of the reaction barriers and coupling constants, we conclude that the studied redox reaction of ML and BL graphene/Cu electrodes is adiabatic, or at best a weakly non-adiabatic reaction. Note that the rate analysis is based on the SNA model Hamiltonian for which all the needed parameters were obtained from constant potential DFT calculations of ML/BL graphene-copper electrodes - no experimental data was used to parametrize the Hamiltonian. The results from our theoretical/computational analysis show that the main difference in the observed ET rates is due to the different electrostatic potentials felt by the redox couple, which, in turn, changes the activation barrier for ET for the adiabatic OS-ET.

Supplementary Note 14: KPFM characterization of characteristic graphene sample on Cu foils

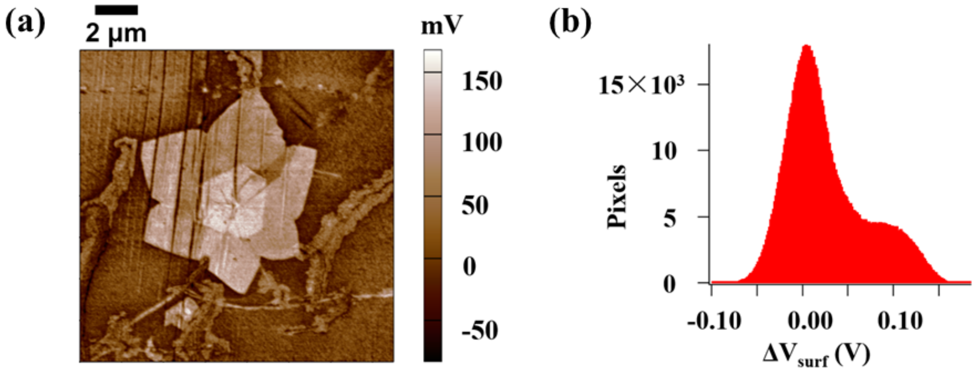

Supplementary Figure 19. (a) Kelvin Probe Force Microscopy (KPFM) measurements provide the surface potential map of graphene used for area 1 and area 2. (b) Histogram distribution of Supplementary Figure 19a. The surface potential of the BL region is around 100 mV higher than that of the ML region.

Supplementary Note 15: DC ion conductance current histogram distributions of area 1

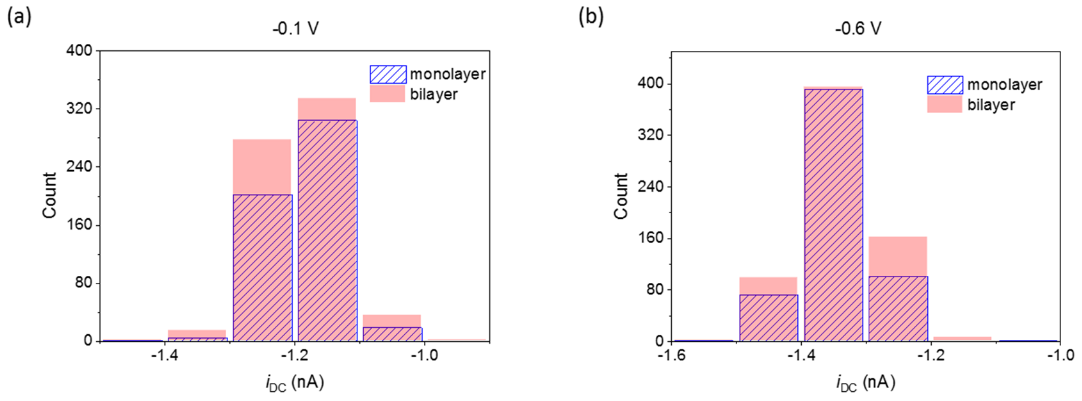

Supplementary Figure 20. DC ion conductance current (i_{DC}) histogram distributions of area 1 at – 0.1 V (a) and -0.6 V (b) vs. Ag/AgCl QRCE in 1 mM $[Ru(NH_3)_6]^{3+}$ (chloride salt).

Supplementary References

1 Ferrari, A. C.; Meyer, J. C.; Scardaci, V.; Casiraghi, C.; Lazzeri, M.; Mauri, F.; Piscanec, S.; Jiang, D.; Novoselov, K. S.; Roth, S.; Geim, A. K. Raman spectrum of graphene and graphene layers. *Phys. Rev. Lett.* **2006**, *97*, 187401.

2 Gupta A.; Chen, G.; Joshi, P.; Tadigadapa, S.; Eklund, P. Raman scattering from high-frequency phonons in supported n-graphene layer films. *Nano Lett.* **2006**, *6*, 2667-2673.

3 Güell, A. G.; Cuharuc, A. S.; Kim, Y.-R.; Zhang, G.; Tan, S.-Y.; Ebejer, N.; Unwin, P. R. Redox-dependent spatially resolved electrochemistry at graphene and graphite step edges. *ACS Nano* **2015**, *9*, 3558-3571.

4 Wang, Y.; Tong, S. W.; Xu, X. F.; Özyilmaz, B.; Loh, K. P. Interface engineering of layer-by-layer stacked graphene anodes for high-performance organic solar cells. *Adv. Mater.* **2011**, *23*, 1514-1518.

5 Güell, A. G.; Ebejer, N.; Snowden, M. E.; Macpherson, J. V.; Unwin, P. R. Structural correlations in heterogeneous electron transfer at ML and multilayer graphene electrodes. *J. Am. Chem. Soc.* **2012**, *134*, 7258-7261.

6 Kear, G. W.; F.; Barker, D.; Stokes, K. The electrochemical corrosion characteristics of copper in filtered and artificial seawater as a function of mass transfer conditions. *EuroCorr*. **2000**, 10-14 .

7 Lee, H.; Nobe, K. Kinetics and mechanisms of Cu electrodissolution in chloride media. *J. Electrochem. Soc.* **1986**, *133*, 2035-2043.

8 Snowden, M. E.; Güell, A. G.; Lai, S. C.; McKelvey, K.; Ebejer, N.; O'Connell, M. A.; Colburn, A. W.; Unwin, P. R. Scanning electrochemical cell microscopy: Theory and experiment for quantitative high resolution spatially-resolved voltammetry and simultaneous ion-conductance measurements. *Anal. Chem.* **2012**, *84*, 2483-2491.

9 Payne, N. A.; Mauzeroll, J. Identifying Nanoscale Pinhole Defects in Nitroaryl Layers with Scanning Electrochemical Cell Microscopy. *ChemElectroChem* **2019**, *6*, 5439-5445.

10 Macpherson, J. V.; Jones, C. F; Unwin, P. R. Radial flow microring electrode: investigation of fast heterogeneous electron-transfer processes. *J. Phys. Chem. B* **1998**, *102*, 9891-9897.

11 Blum, V.; Gehrke, R.; Hanke, F.; Havu, P.; Havu, V.; Ren, X.; Reuter, K.; Scheffler, M. Ab initio molecular simulations with numeric atom-centered orbitals. *Comput. Phys. Commun.* **2009**, *180*, 2175-2196.

12 Blöchl, P. E. Projector augmented-wave method. *Phys. Rev. B* **1994**, *50*, 17953.

13 Mortensen, J. J., Hansen, L. B.; Jacobsen, K. W. Real-space grid implementation of the projector augmented wave method. *Phys. Rev. B* **2005**, *71*, 035109.

14 Enkovaara, J. et al. Electronic structure calculations with GPAW: a real-space implementation of the projector augmented-wave method. *J. Condens. Matter Phys.* **2010**, *22*, 253202.

15 Larsen, A. H. et al. The atomic simulation environment—a Python library for working with atoms. *J. Condens. Matter Phys.* **2017**, *29*, 273002.

16 Ruiz, V. G.; Liu, W.; Zojer, E.; Scheffler, M.; Tkatchenko, A. Density-functional theory with screened van der Waals interactions for the modeling of hybrid inorganic-organic systems. *Phys. Rev. Lett.* **2012**, *108*, 146103.

17 Larsen, A. H.; Vanin, M.; Mortensen, J. J.; Thygesen, K. S.; Jacobsen, K. W. Localized atomic basis set in the projector augmented wave method. *Physical Review B* **2009**, *80*, 195112.

18 Perdew, J. P.; Burke, K.; Ernzerhof, M. Generalized gradient approximation made simple. *Phys. Rev. Lett.* **1996**, *77*, 3865.

19 Melander, M. M.; Kuisma, M. J.; Christensen, T. E. K.; Honkala, K. Grand-canonical approach to density functional theory of electrocatalytic systems: Thermodynamics of solid-liquid interfaces at constant ion and electrode potentials. *J. Chem. Phys.* **2019**, *150*, 041706.

20 Kastlunger, G.; Lindgren, P.; Peterson, A. A. Controlled-potential simulation of elementary electrochemical reactions: Proton discharge on metal surfaces. *J. Phys. Chem. C* **2018**,

122, 12771-12781.
21 Held, A.; Walter, M. Simplified continuum solvent model with a smooth cavity based on volumetric data. *J. Chem. Phys.* **2014**, *141*, 174108.
22 Łukomska, A.; Sobkowski, J. Potential of zero charge of monocrystalline copper electrodes in perchlorate solutions. *J. Electroanal. Chem.* **2004**, *567*, 95-102.
23 Trasatti, S. The “absolute” electrode potential—The end of the story. *Electrochim. Acta* **1990**, *35*, 269-271.
24 Cheng, J.; Sprik, M. Alignment of electronic energy levels at electrochemical interfaces. *Phys. Chem. Chem. Phys.* **2012**, *14*, 11245-11267.
25 Newton, M. D. Quantum chemical probes of electron-transfer kinetics: the nature of donor-acceptor interactions. *Chem. Rev.* **1991**, *91*, 767-792.
26 Anderson, P. W. Localized magnetic states in metals. *Phys. Rev.* **1961**, *124*, 41.
27 Newns, D. Self-consistent model of hydrogen chemisorption. *Phys. Rev.* ***1969***, *178*, 1123.
28 Schmickler, W. A theory of adiabatic electron-transfer reactions. *J. Electroanal. Chem. and intet. Electrochem.* **1986**, *204*, 31-43.
29 Schmickler, W. Adiabatic and non-adiabatic electrochemical electron transfer in terms of Green’s function theory. *Russ. J. Electrochem.* **2017**, *53*, 1182-1188.
30 Huang, J.; Chen, S. Interplay between covalent and noncovalent interactions in electrocatalysis. *J. Phys. Chem. C* **2018**, *122*, 26910-26921.
31 Lam, Y. C.; Soudackov, A. V.; Goldsmith, Z. K.; Hammes-Schiffer, S. Theory of Proton Discharge on Metal Electrodes: Electronically Adiabatic Model. *J. Phys. Chem. C* **2019**, *123*, 12335-12345 .
32 Smalley, J. F.; Finklea, H. O.; Chidsey, C. E. D.; Linford, M. R.; Creager, S. E.; Ferraris J. P.; Chalfant, K.; Zawodzinsk, T.; Feldberg, S. W.; Newton, M. D. Heterogeneous electron-transfer kinetics for ruthenium and ferrocene redox moieties through alkanethiol MLs on gold. *J. Am. Chem. Soc.* **2003**, *125*, 2004-2013.
33 Nazmutdinov, R. R.; Berezin, A. S.; Soldano, G.; Schmickler W. Orbital overlap effects in electron transfer reactions across a metal nanowire/electrolyte solution interface. *J. Phys. Chem. C* **2013**, *117*, 13021-13027.
34 Pavlov, S. V.; Kislenko, V. A.; Kislenko, S. A. Fast Method for Calculating Spatially Resolved Heterogeneous Electron-Transfer Kinetics and Its Application to Graphene with Defects. *J. Phys. Chem. C* **2020**, *124*, 18147-18155.
