# Supplementary material for: Adiabatic versus non-adiabatic electron transfer at 2D electrode materials
Source: Nat Commun. 2021 Dec 7;12:7110. doi: 10.1038/s41467-021-27339-9 (PMC8651748; doi:10.1038/s41467-021-27339-9)
Supplement: Supplementary file 3 — Description of Additional Supplementary Files [file 41467_2021_27339_MOESM3_ESM.pdf]

Description of Additional Supplementary Files

File Name: Supplementary Movie 1
Description: Current as a function of potential for area 1

File Name: Supplementary Movie 2
Description: Current as a function of potential for area 2
